# Transcutaneous auricular vagus nerve stimulation to alleviate metformin-associated gastrointestinal adverse events and optimize glycaemic control: a randomized, sham-controlled pilot trial protocol

**DOI:** 10.3389/fnins.2026.1744208

**Published:** 2026-04-17

**Authors:** Xiaolei Ge, Zhengrong Luo, Youwen Zhang, Lu Zhang, Ge Wang, Xinyi Yuan, Qian Li, Xu Zhai

**Affiliations:** 1 Beijing Hospital of Traditional Chinese Medicine, Capital Medical University, Digestive Disease Diagnosis and Treatment Center, Dongcheng, Beijing, China; 2 Wangjing Hospital, China Academy of Chinese Medical Sciences, Chaoyang, Beijing, China; 3 Institute of Chinese Medical History and Literature, China Academy of Chinese Medical Sciences, Dongcheng, Beijing, China; 4 Graduate School, China Academy of Chinese Medical Sciences, Dongcheng, Beijing, China; 5 Xiyuan Hospital, China Academy of Chinese Medical Sciences, Haidian, Beijing, China; 6 Jinan Hospital of Guang'anmen Hospital, China Academy of Chinese Medical Sciences, Jinan, Shandong, China

**Keywords:** gastrointestinal intolerance, metformin, randomized controlled trial, transcutaneous auricular vagus nerve stimulation, type 2 diabetes mellitus

## Abstract

**Background:**

Gastrointestinal adverse events (GI AEs) are the main dose-limiting side effects of metformin in type 2 diabetes mellitus (T2DM), reducing adherence and compromising long-term glycaemic control. Current strategies (dose adjustment or combination therapy) seldom address both tolerability and sustained metabolic efficacy. Transcutaneous auricular vagus nerve stimulation (taVNS) is a non-invasive neuromodulation technique that may modulate gut–brain–metabolic pathways—vagal reflexes, inflammation, intestinal barrier function, and enteroendocrine signaling—and thus improve drug tolerance while preserving glycaemic control.

**Methods:**

This single-center, randomized, sham-controlled pilot trial will enroll 60 T2DM patients with metformin-associated GI AEs, randomized 1:1 to either the taVNS group or the sham control group. The intervention lasts 2 weeks with a follow-up at week 4. Assessments at baseline and follow-up include a validated Metformin Symptom Severity Score (total score 0–50; primary outcome), Bristol Stool Form Scale, bowel urgency, glycaemic/metabolic indices [fasting blood glucose (FBG), 2-h postprandial glucose (PG2h), glycated albumin (GA), fasting C-peptide, fasting insulin, HOMA-IR, ISI], and mechanistic biomarkers (GLP-1, 5-HT, IL-6, IL-10, TNF-α, D-lactate, DAO, bile acids). Safety monitoring includes routine hematology, liver and renal function tests.

**Discussion:**

By combining clinical outcomes with targeted biomarker analyses in a randomized design, this pilot study will assess whether taVNS alleviates metformin-associated GI intolerance without impairing glycaemic efficacy, and will provide feasibility data, effect-size estimates, and biomarker selection for future confirmatory trials.

**Clinical trial registration:**

Trial registration International Traditional Medicine Clinical Trial Registry (ITMCTR) http://itmctr.ccebtcm.org.cn/, Identifier: ITMCTR2025001086.

## 1 Introduction

Type 2 diabetes mellitus (T2DM), characterized by insulin resistance and impaired insulin secretion, is increasing in prevalence and constitutes a major global public-health challenge (Giaccari et al., 2012). Metformin is universally recommended as first-line therapy in contemporary diabetes guidelines because of its favorable safety profile, cost-effectiveness, and demonstrated long-term safety—a recommendation supported by several high-quality meta-analyses (Maruthur et al., 2016; Palmer et al., 2016). Emerging evidence further indicates that, beyond glycaemic control, metformin exerts pleiotropic effects including cardiovascular protection and antineoplastic actions (Rena et al., 2013; Rojas and Gomes, 2013).

However, gastrointestinal adverse events (GI AEs) are the most common dose-limiting side effect of metformin and substantially impair treatment adherence and long-term outcomes. Reported non-adherence to metformin reaches 43%, notably higher than rates observed for statins (36%) or angiotensin-converting enzyme inhibitors (23%) (Pladevall et al., 2004). In a randomized study of over 1,400 patients with T2DM, 23.7% of participants assigned to metformin experienced diarrhea, 15.4% reported abdominal discomfort, 11.7% reported nausea, and 5.8% experienced vomiting—rates higher than in the glibenclamide or rosiglitazone arms (Kahn et al., 2006). These adverse effects not only undermine adherence but also substantially diminish quality of life (Pladevall et al., 2004; Florez et al., 2010). Although dose reduction can improve tolerability, it concomitantly attenuates dose-dependent glycaemic efficacy (optimal effects are typically observed at doses approaching 2,000 mg/day), underscoring the clinical value of interventions that preserve both tolerability and efficacy (Garber et al., 1997).

Mechanistically, metformin-related GI AEs center on drug–gut interactions. Oral bioavailability of metformin is only ~50–60%, yet its antihyperglycaemic effects often exceed those of intravenous administration (Stepensky et al., 2002), and intestinal mucosal concentrations are markedly higher than plasma levels (Bailey et al., 1994; Mccreight et al., 2016)—all indicating that the gut is a principal site of action. Metformin stimulates L-cell secretion of glucagon-like peptide-1 (GLP-1) and may weakly inhibit dipeptidyl peptidase-4 (DPP-4), thereby increasing active GLP-1; elevated GLP-1 can delay gastric emptying and alter intestinal transit (Migoya et al., 2010). Structurally similar to certain 5-HT3 receptor agonists, metformin can promote enterochromaffin 5-hydroxytryptamine (5-HT) release and may impair 5-HT uptake by inhibiting organic cation transporters (OCT1, OCT3) and the serotonin transporter (SERT), leading to increased 5-HT availability and altered gut motility (Yee et al., 2015; Mawe and Hoffman, 2013). Metformin also perturbs bile acid handling by reducing intestinal bile salt hydrolase activity and increasing ileal bile flow, which can impair bile acid reabsorption and, through bile acid–microbiota interactions, further modulate GLP-1 secretion (Carter et al., 2003; Beysen et al., 2012). Additionally, the gut is a major site of lactate production; metformin raises intestinal lactate concentrations [confirmed in human jejunal biopsies (Mccreight et al., 2016)] and inhibits hepatic mitochondrial glycerol-3-phosphate dehydrogenase, reducing lactate clearance and promoting local accumulation that may directly irritate the mucosa (Bailey et al., 1992; Madiraju et al., 2014). Reduced OCT1 activity or genetic polymorphisms can increase intraluminal metformin accumulation, exacerbate anaerobic metabolism, and augment lactate generation (Singh et al., 2023; Dujic et al., 2016). Barrier dysfunction and ensuing inflammatory mediators, together with altered levels of GLP-1, 5-HT, IL-6, IL-10, TNF-α, D-lactate, diamine oxidase (DAO), and bile acids, contribute to dysbiosis—reported increases in taxa such as Akkermansia spp. and certain γ-Proteobacteria have been observed in T2DM patients (Shin et al., 2014)—and dysbiosis may in turn promote symptomatology by increasing pathogenic factor release and disrupting bile-acid metabolism (Ramos et al., 2016).

Because adherence is inversely associated with glycaemic control and directly associated with reduced healthcare costs (Lawerence et al., 2006), sustained metformin use is critical for disease management. Given the dose-dependent nature of metformin's efficacy [with optimal glycaemic effects observed near 2,000 mg/day (Garber et al., 1997)], attenuating GI AEs without reducing dose has important implications for treatment outcomes. Current clinical management largely relies on dose adjustment or adjunctive symptomatic therapies and lacks a systematic, mechanism-based “enhance-efficacy/reduce-toxicity” strategy rooted in the gut–brain–metabolic axis.

Against this pathophysiological background, non-invasive vagal modulation via transcutaneous auricular vagus nerve stimulation (taVNS) offers a novel therapeutic perspective. taVNS selectively activates the auricular branch of the vagus nerve (ABVN) and, through an ear–vagus–brainstem–gut circuit (Zou et al., 2026), can modulate central-peripheral autonomic balance, engage cholinergic anti-inflammatory pathways, regulate gut motility, influence endocrine mediators (including GLP-1 and 5-HT), and affect bile-acid metabolism, while indirectly reshaping the gut microbiota and barrier function (The Wingate Institute of Neurogastroenterology TBI, 2020; Seitz et al., 2022; Zhao et al., 2025; Zhang et al., 2025; Zou et al., 2024). Thus, taVNS could theoretically mitigate local inflammation and functional perturbations induced by metformin without necessitating dose reduction, thereby improving tolerability and synergistically enhancing metabolic control. The ear concha region corresponds anatomically to a dense distribution of ABVN fibers (The Wingate Institute of Neurogastroenterology TBI, 2020); transcutaneous stimulation of this area activates vagal pathways while avoiding the invasiveness of implanted VNS and can influence both gastrointestinal function and glucose metabolism (Zou et al., 2024; He et al., 2013).

Given the multifactorial mechanisms underlying metformin-induced GI AEs, taVNS may exert therapeutic effects through multiple converging pathways: modulation of the gut microbiota (e.g., reduction of certain opportunistic taxa) and bile-acid metabolism (Zhang et al., 2025); down-regulation of proinflammatory cytokines such as TNF-α and IL-6 via vagal-cholinergic anti-inflammatory signaling (Zhao et al., 2025); bidirectional regulation of 5-HT and GLP-1 to counteract metformin-induced elevations (Yang et al., 2026); and reduction of serum D-lactate levels with consequent improvement of mucosal barrier function (Seitz et al., 2022). Importantly, taVNS may also enhance vagal efferent activity and physiologic GLP-1 secretion, thereby complementing metformin's insulin-sensitizing actions and supporting improved glycaemic management (Yee et al., 2015).

It should be emphasized that traditional ear-acupoint localization (for example, spleen and colon regions in auricular acupuncture) provides historical and anatomical rationale for stimulation site selection, but mechanistic validation requires imaging, electrophysiological, and molecular evidence. Overall, taVNS presents a testable mechanistic hypothesis to resolve the “enhance-efficacy/reduce-toxicity” dilemma of metformin and merits systematic evaluation in randomized clinical and translational studies.

## 2 Methods

### 2.1 Study design

This study is designed as a single-center, randomized, sham-controlled clinical trial conducted at Wangjing Hospital, China Academy of Chinese Medical Sciences. The trial aims to evaluate the efficacy of taVNS in alleviating metformin-associated GI AEs and to explore its underlying mechanisms in improving drug tolerance and synergistically enhancing glycaemic control through analyses of molecular, metabolic, and inflammatory biomarkers.

The trial is designed in accordance with the Standard Protocol Items: Recommendations for Interventional Trials (SPIRIT) guidelines. Participant recruitment is expected to commence in December 2025, with completion of primary follow-up anticipated by March 2026. Eligible participants who meet all inclusion and exclusion criteria and provide written informed consent will be randomly assigned (1:1) to either the taVNS intervention group or the sham stimulation control group. The intervention period will last 2 weeks, followed by a 4-week post-treatment follow-up.

The protocol has been approved by the Institutional Ethics Committee (approval number: WIEC-YJS-2025-003-P002). To ensure participant safety and data integrity, an independent Data Monitoring Committee (DMC) has been established. The DMC will conduct periodic reviews of adverse events, trial conduct, and data quality.

**Figure 1:**
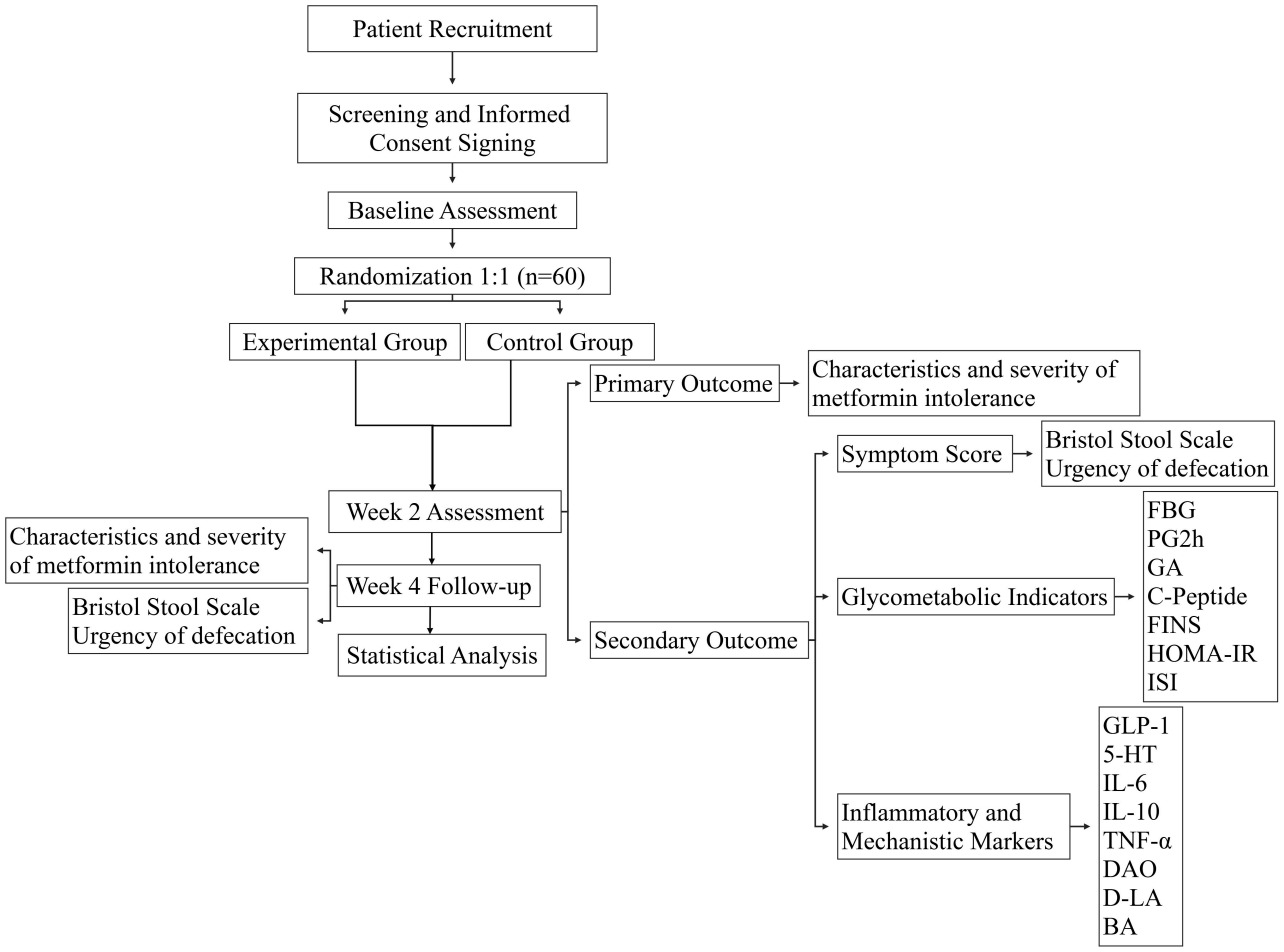
Schematic flow diagram of the study design.

A schematic flow of the study procedure is illustrated in Figure 1.

### 2.2 Recruitment

Participants will be recruited from the outpatient clinics of Wangjing Hospital, China Academy of Chinese Medical Sciences, via hospital posters, internal notices and the hospital's official WeChat account. The study team will screen potential participants against the predefined inclusion and exclusion criteria. Only individuals who meet all eligibility criteria and who provide written informed consent will be enrolled. All study data will be managed by authorized personnel; access will be restricted to designated, authorized members of the study team and the independent DMC. Personally identifiable information will be kept strictly confidential and stored in encrypted form in accordance with applicable institutional and national data-protection regulations.

### 2.3 Diagnostic criteria

#### 2.3.1 Diagnostic criteria for type 2 diabetes mellitus

The diagnosis of diabetes will follow World Health Organization (WHO) criteria (Puavilai et al., 1999; Kansagara et al., 2019). A diagnosis of T2DM will be made if any of the following criteria are satisfied:

(1) Fasting plasma glucose (FPG) ≥ 126 mg/dl (7.0 mmol/L) after an overnight fast of at least 8–14 h (venous or capillary);
(2) 2-h postprandial venous plasma glucose ≥ 200 mg/dl (11.1 mmol/L);
(3) 2-h postprandial capillary plasma glucose ≥ 220 mg/dl (12.2 mmol/L);
(4) Random plasma glucose ≥ 200 mg/dl (11.1 mmol/L);
(5) Glycated hemoglobin (HbA1c) ≥ 6.5%.

Borderline or inconclusive results should be repeated on a subsequent day. Typical hyperglycaemic symptoms (for example, polyuria, polydipsia, unexplained weight loss, or blurred vision) may support the diagnosis.

#### 2.3.2 Diagnostic criteria for metformin intolerance

Metformin intolerance will be defined as meeting any of the following criteria (McCreight et al., 2018):

(1) Prior use of metformin with treatment discontinuation due to gastrointestinal intolerance (prior exposure ≤ 1,000 mg/day for ≤ 8 weeks); or
(2) Inadequate glycaemic control [e.g., HbA1c ≥ 7.0% (53 mmol/mol)] with inability to escalate metformin to ≥500 mg/day because of intolerable gastrointestinal symptoms.

(Reference tolerability: individuals taking divided doses of metformin up to 2,000 mg/day without clinically significant gastrointestinal adverse effects.) Other causes of diarrhea must be excluded before confirming the diagnosis (McCreight et al., 2018).

#### 2.3.3 Inclusion criteria

Participants must meet all of the following:

(1) Fulfill the diagnostic criteria for T2DM and for metformin intolerance as defined above;
(2) Aged 18–80 years (inclusive), any sex;
(3) Able and willing to comply with study procedures and to provide written informed consent;
(4) Stable diet, exercise, lifestyle and antihyperglycaemic regimen, with a fasting blood glucose coefficient of variation (CV) < 16% during the 3 days preceding baseline and on the day of enrollment;
(5) Body mass index (BMI) between 18.5 and 40.0 kg/m^2^.

Only participants who satisfy all five criteria will be enrolled.

#### 2.3.4 Exclusion criteria

Subjects meeting any of the following will be excluded:

(1) Acute or chronic diarrhea attributable to confirmed gastrointestinal pathology or systemic disease (including but not limited to hepatic, biliary or pancreatic disease; infectious diarrhoeal illnesses such as dysentery or cholera; systemic diseases, intoxications, parasitic infections; malignancy; prior gastric bypass surgery);
(2) Severe cardiovascular or cerebrovascular disease, diabetic ketoacidosis, or other serious endocrine, haematologic or psychiatric disorders likely to affect compliance or follow-up;
(3) Use of antibiotics, proton pump inhibitors, H_2_-receptor antagonists, probiotics/prebiotics or other agents known to substantially alter the gut microbiota within 4 weeks prior to enrollment;
(4) Pregnancy, planned pregnancy, or lactation;
(5) Marked glycaemic instability;
(6) Current insulin therapy;
(7) Severe skin lesions in the auricular concha or absence/deformity of the auricle that precludes intervention;
(8) Fear of the intervention or inability to tolerate conventional electrical stimulation;
(9) Known allergy or contraindication to metformin hydrochloride;
(10) Participation in another clinical trial or any condition likely to prevent completion of the study follow-up.

Note: any participant who meets one or more of the exclusion criteria above will be ineligible for enrollment.

### 2.4 Sample size justification

Sample size for this randomized, sham-controlled pilot and feasibility trial was determined in full alignment with the CONSORT 2010 Statement: Extension to Randomized Pilot and Feasibility Trials (Eldridge et al., 2016), and the optimized sample size estimation method for pilot trials with continuous primary outcomes established by Whitehead et al (Whitehead et al., 2016). As an external pilot trial, the core aim of sample size setting is not to power formal hypothesis testing for intervention efficacy, but to generate a sufficiently precise estimate of the standard deviation of our primary outcome (Metformin Symptom Severity Score total score) to inform the design of a future definitive randomized controlled trial. In detail, according to the optimized sample size estimation method for pilot trials established by Whitehead et al., an estimated final sample of 25 evaluable participants per group (after accounting for a 15% attrition rate from the 30 enrolled participants) yields 48 degrees of freedom for variance estimation. This well exceeds the minimum threshold of 20 degrees of freedom recommended in the literature for stable, unbiased variance estimation in pilot research. No formal hypothesis testing for intervention efficacy is planned in this pilot trial. Core feasibility endpoints include: recruitment rate (target ≥8 participants per month), screening failure rate, treatment adherence rate (defined as completing ≥80% of scheduled treatment sessions), attrition rate (estimated at 15% based on published neuromodulation trials in patients with T2DM), and protocol violation rate. Accounting for a 15% attrition rate, the final enrollment target is 60 participants (30 per group) (Whitehead et al., 2016).

### 2.5 Randomization and blinding

Participants will be randomized 1:1 to taVNS group or sham control group, with stratification by age and gender to balance baseline characteristics between groups. The randomization sequence will be generated by an independent third-party biostatistician using SPSS 26.0, with group allocation sealed in sequentially numbered, opaque envelopes for strict allocation concealment. After baseline assessment, a dedicated study coordinator (fully blinded to group assignment) will open envelopes in numerical order to assign eligible participants.

Given the anatomical specificity of active and sham stimulation sites, intervention staff responsible for electrode placement and device operation cannot be blinded to group allocation. To mitigate potential bias, we have implemented pre-specified rigorous safeguards: The blinded coordinator exclusively manages group allocation, and only provides intervention staff with pre-marked anatomical diagrams of stimulation sites, with no disclosure of group identity at any time. Non-blinded intervention staff are strictly barred from outcome assessment, data entry, statistical analysis, or any communication with participants about group allocation or expected intervention effects. Both groups use identical stimulators with consistent nominal parameters; all intervention staff receive centralized standardized training to ensure identical operational execution and participant interaction across groups.

All outcome assessors, data analysts, biostatisticians, and participants will remain fully blinded to group allocation throughout the trial. Furthermore, treating physicians are solely responsible for routine clinical management and safety assessments; they have no access to group allocation and are strictly isolated from intervention implementation and outcome assessment. Participants will be informed that both interventions may induce a mild tingling sensation, and subjective feeling cannot be used to infer group assignment, to minimize anticipation bias. Outcome assessors will receive standardized training and regular inter-rater reliability checks to reduce measurement bias.

The effectiveness of the blinding will be formally assessed in Weeks 2 and 4 using the validated James Blinding Index (BI) method (James et al., 1996). The detailed statistical procedures for this assessment are outlined in the “Statistical Methods” section.

### 2.6 Interventions

This is a parallel-group trial employing a “standard care + targeted intervention” design. Both groups will receive standardized diabetes management and metformin therapy as background treatment; in addition, participants will receive either taVNS or sham control group. The treatment course for both arms is 2 weeks, with subsequent follow-up assessments.

#### 2.6.1 Standard care (background therapy)

Background therapy follows international guideline recommendations and comprises standardized lifestyle counseling and medication management. Lifestyle counseling includes: dietary guidance with carbohydrates accounting for 45–60% of total daily energy intake and dietary fiber ≥25 g/day; at least 150 min/week of moderate-intensity aerobic activity (for example, brisk walking or cycling); and standardized education on disease management, medication adherence, and management of gastrointestinal symptoms to ensure consistency across participants (American Diabetes Association, 2024a,b).

Medication management principles are as follows:

Discontinue or avoid medications known to exacerbate gastrointestinal symptoms where clinically appropriate (e.g., stimulant laxatives or certain over-the-counter preparations);

In participants with inadequate glycaemic control, additional antihyperglycaemic agents (for example, SGLT2 inhibitors or DPP-4 inhibitors) may be added as clinically indicated, provided such additions are unlikely to aggravate gastrointestinal function; adjustments will follow current guideline recommendations and be documented;

Concomitant treatment for chronic conditions (e.g., antihypertensives for hypertension or secondary prevention therapies for coronary artery disease) will be maintained but clinicians should avoid introducing agents known to provoke GI intolerance when alternatives are available.

Metformin dosing will follow a “start low, escalate gradually” regimen: initial dose 500 mg administered with meals twice daily; dose titration will proceed weekly guided by fasting blood glucose (target 4.4–7.0 mmol/L), with a maximum dose up to 2,000 mg/day or the participant's maximal tolerated dose. Dose adjustments and all concomitant medications will be supervised and recorded by study physicians. In the event of severe, poorly tolerated diarrhea (e.g., ≥5 bowel motions per day with clinical signs of dehydration), metformin will be withheld or adjusted and symptomatic therapy provided (e.g., diosmectite); severe events will be managed according to the study Serious Adverse Event (SAE)—defined per the Common Terminology Criteria for Adverse Events (CTCAE) as any adverse event resulting in death, life-threatening illness, hospitalization, persistent disability, or requiring immediate emergency intervention—procedure and continue to be followed up (Rena et al., 2013).

To ensure ethical fairness, control-group participants who meet predefined criteria at study end may be offered compensatory taVNS treatment if clinically appropriate; this arrangement will be described in the informed consent form.

#### 2.6.2 Intervention arm (taVNS)

**Figure 2:**
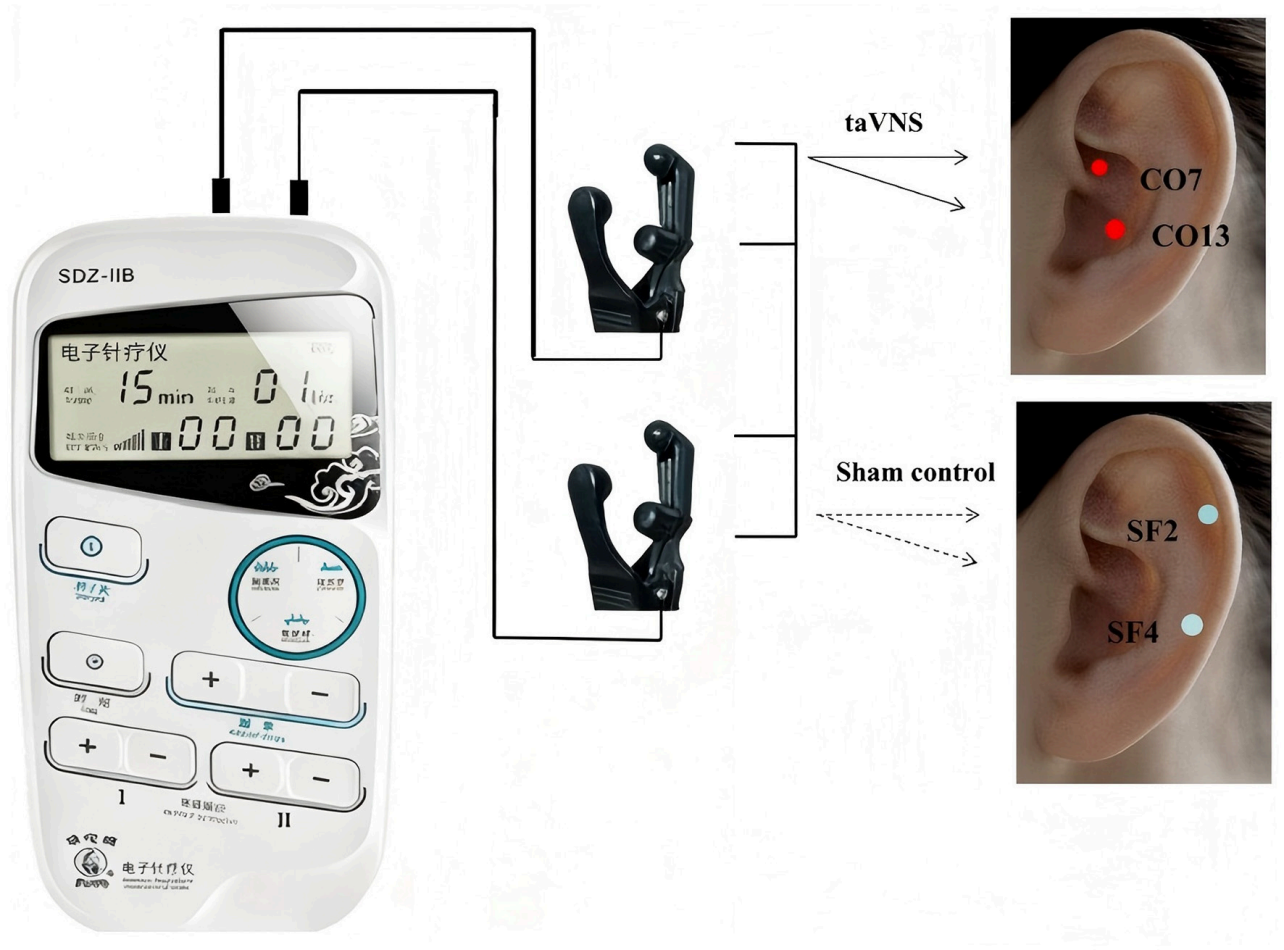
Device and stimulation sites. Both groups used the HuaTuo SDZ-IIB device with identical parameters. Red dots indicate taVNS sites: CO7 (large intestine point) and CO13 (spleen point) in the cymba conchae. Blue dots indicate sham sites: SF2 (wrist point) and SF4 (shoulder point) in the scapha.

In addition to standard care, participants randomized to the intervention arm will receive taVNS delivered using a Huatuo SDZ-IIB electronic acupuncture stimulator (see Figure 2). Procedures are as follows:

Stimulation site: auricular regions with high density of auricular branch vagus nerve (ABVN) fibers—specifically the cymba conchae and cavum conchae—targeting auricular “Large Intestine Point” (CO7) and “Spleen Point” (CO13) points as anatomical targets for modulation of gastrointestinal function.

Preparation: prior to each stimulation session, trained personnel will disinfect the skin, place and secure electrodes over the marked sites, and verify electrode impedance and device function.

Stimulation parameters: intermittent stimulation with an on/off cycle of 2 s on/3 s off; pulse width of 0.5 ms; frequency approximately 25 Hz; and current intensity individualized within the range 0.5–1.5 mA, titrated to achieve a mild tingling or warm sensation without pain. Intensity will be adjusted according to participant tolerance. This treatment protocol is based on a multicenter randomized controlled trial protocol validated for gastrointestinal disorders (Zhu et al., 2021; Shi et al., 2024; Yin, 2025). Furthermore, evidence from Molefi et al. (2025), which examined healthy subjects with visual-induced motion sickness and found that a 20 Hz frequency combined with a 0.2 ms pulse width did not significantly improve the composite gastrointestinal symptom score, further supporting the rationale for our choice of parameters.

Adverse sensations: if a participant experiences notable discomfort during stimulation, the intensity will be reduced or the session terminated and the event recorded as an adverse event (AE).

Detailed session duration and scheduling will follow the study protocol (see Figure 2 and the procedure manual), and all interventions will be administered by personnel trained in taVNS application.

#### 2.6.3 Control arm (sham)

The control arm will receive a sham stimulation designed to control for non-specific effects of device application and patient interaction while avoiding stimulation of vagal auricular sites. Procedures mirror those of the intervention arm with the following distinctions:

Stimulation site: electrodes will be placed on the scapha (non-vagal area), targeting auricular “Shoulder Point” (SF4) and “Wrist Point” (SF2) points that are not anatomically associated with ABVN or gastrointestinal vagal pathways, ensuring no anatomical overlap with the active taVNS targets (Peuker and Filler, 2002).

Device and procedures: the identical Huatuo SDZ-IIB device model, electrode preparation, disinfection, participant positioning, and nominal stimulation parameter settings (pulse pattern, frequency, and nominal current range) will be used to preserve blinding. The only intentional difference is the stimulation target (site); internal device output routing will be arranged so that the sham site receives a non-vagal stimulation pattern while maintaining similar sensation characteristics.

This sham control procedure is intended to preserve participant blinding and to isolate the specific effects of auricular vagal stimulation.

Notes on safety and documentation.

All interventions, device settings, session timing, and any deviations will be documented in the case report form (CRF). Adverse events will be recorded and managed according to the protocol's safety monitoring plan and reported to the DMC and ethics committee per predefined timelines.

#### 2.6.4 Treatment schedule and operational management

All participants will receive two stimulation sessions per day (morning and evening), each lasting 30 min, on 5 consecutive weekdays (Monday–Friday) for 2 consecutive weeks (total 10 sessions). During the initial treatment phase, the first three sessions will be administered and supervised in hospital outpatient or inpatient settings by trained study personnel; these sessions also serve as hands-on training for participants who will continue treatment at home. Thereafter, participants may complete remaining sessions at home according to the training protocol.

Home-based treatment requires daily completion of a treatment log documenting treatment time, device current intensity, local sensations, and any gastrointestinal symptoms. Study staff will review logs and confirm adherence weekly by telephone or via a secure remote platform, and will address participant queries. All interventions and any medication adjustments will be performed according to standard operating procedures (SOPs), recorded contemporaneously, and entered into the case report form (CRF).

Any deviation from the prescribed stimulation parameters or protocol procedures must be recorded with rationale. All staff administering or supervising treatments will be trained and certified according to the study training curriculum; competency assessments will be documented.

#### 2.6.5 Observation schedule and sample handling

The overall observation period is 5 weeks, comprising a 1-week baseline period (Week −1 to 0), a 2-week treatment period (Weeks 0–2), and a 2-week follow-up period (Weeks 2–4). Primary and key secondary outcomes (Metformin Symptom Severity Score, Bristol Stool Form Scale, and bowel urgency) as well as glycaemic/metabolic indices and selected inflammatory and mechanistic biomarkers will be assessed at baseline, Week 2, and Week 4. Selected biochemical markers will be repeated at baseline and either Week 2 or Week 4 according to the prespecified sampling schedule in the protocol appendix.

All biological specimen collection, processing, transport, and storage will follow a unified SOP. Blood samples will be drawn after an overnight fast unless otherwise specified, kept short-term at 4 °C, and transferred to long-term storage at −80 °C within the time limits defined in the SOP. Laboratory assay methods, quality-control procedures, and batch management are described in the trial appendix. The timeline of interventions, assessments, and visit windows is detailed in Table 1; adverse-event monitoring will be continuous throughout the entire study period.

**Table 1:** Treatment and data collection.

Category	Study phase	Baseline	Visit 1	Visit 2
	Visit window	(Day 0)	Week 2	Week 4
Information collection	Basic medical history collection	×		
	Informed consent obtained	×		
	General demographics collection	×		
	Past medical and treatment history	×		
	Comorbidities and treatment history	×		
	Eligibility criteria check	×		
Primary outcomes	Metformin symptom severity score	×	×	×
Secondary outcomes	Bristol stool form scale	×	×	×
	Defecation urgency	×	×	×
	Quality of life scale	×	×	×
	FBG	×	×	×
	PG2h	×	×	×
	GA	×	×	
	IL-6, IL-10, TNF-α		×	
	GLP-1, BA, 5-HT, D-LA		×	
	ALT, AST, BUN, Cr (liver and renal function)		×	
Sample and data management	Adverse events (AEs) monitoring		×	×
	Sample collection record		×	
	CRF review by investigator (CRF-PI)	×	×	×
	CRF review by monitor (CRF-MON)	×	×	×
	Blinding assessment		×	×

“ × ” indicates that the corresponding assessment is performed at that visit. Baseline = day 0; visit 1 = week 2 (±3 days); visit 2 = week 4 (±3 days). All blood and stool specimens will be collected after an overnight fast.

### 2.7 Outcome measures and time points

#### 2.7.1 General assessments

Demographic and medical history data, including height, weight, sex, age, BMI, prior cholecystectomy, duration of diabetes, current glucose-lowering regimen, comorbid treatments, alcohol/smoking history, allergy history, and family history, will be collected at baseline. These data will be used to control for confounders and perform subgroup analyses.

#### 2.7.2 Primary efficacy outcome and assessment time points

The primary efficacy endpoint is the total score of the Metformin Symptom Severity Score, a validated, widely used self-report tool originally developed and psychometrically validated by McCreight et al. specifically for evaluating metformin-related gastrointestinal intolerance in adults with T2DM (McCreight et al., 2018).

In the original validation study by McCreight et al., this questionnaire demonstrated excellent reliability and validity: it showed strong internal consistency (Cronbach's α > 0.8), good test-retest reliability, and significant discriminant validity, with a highly significant difference in total scores between metformin-intolerant and metformin-tolerant patients (*P* < 0.0001) (McCreight et al., 2018). The questionnaire covers medication adherence, reasons for discontinuation, dose tolerance, gastrointestinal symptoms (nausea, abdominal bloating/pain, and diarrhea; frequency and subjective severity), changes in baseline symptoms, and an overall subjective tolerance rating, with a total score ranging from 0 to 50 points (0–10 = tolerable, 11–20 = mild intolerance, 21–30 = intolerable, 31–50 = severe intolerance). This questionnaire is used for the quantitative assessment of intolerance-related symptoms in this trial, not as a basis for clinical diagnosis.

Assessment time points include baseline, Week 2, and Week 4. Details of the questionnaire items and scoring criteria are provided in Table 2 (McCreight et al., 2018).

**Table 2:** The metformin symptom severity score questionnaire.

No	Question	Option	Score
1	Are you still taking metformin?	Yes (skip to question 6)	0
		No	5
2	If you have stopped taking metformin, was it due to side effects?	Yes (continue to question 3)	5
		No (skip to question 6)	0
3	What dose were you taking at the time of discontinuing metformin?	500 mg once daily (1 tablet)	4
		1,000 mg once daily (2 tablets)	3
		500 mg twice daily (2 tablets)	2
		2,000 mg once daily (4 tablets)	1
		1,000 mg twice daily (2 tablets)	0
4	Have you ever taken a lower dose of metformin and tolerated it well?	Yes	0
		No	1
	If yes, what was the maximum dose you tolerated?	(e.g., 500 mg once daily)	2
5	Have you been trialed on a modified release preparation of metformin (e.g. Glucophage SR)?	Yes	2
		No	0
In the last week of taking metformin, have you experienced?			
6	Nausea?	Yes	1
		No	0
	If yes, how often?	More than once a day	4
		Once a day	3
		Occasionally	2
		Only once	1
	How would you score its severity from 1 to 5 (where 1 is mild, not causing distress or affecting your daily routine, and 5 is very severe, causing marked distress and/or disrupting your daily activities)?	_______/5	
7	Abdominal bloating/pain?	Yes	1
		No	0
	If yes, how often?	More than once a day	4
		Once a day	3
		Occasionally	2
		Only once	1
	How would you score its severity from 1 to 5 (where 1 is mild, not causing distress or affecting your daily routine, and 5 is very severe, causing marked distress and/or disrupting your daily activities)?	_______/5	
8	Diarrhea?	Yes	1
		No	0
	If yes, how often?	More than once a day	4
		Once a day	3
		Occasionally	2
		Only once	1
	How would you score its severity from 1 to 5 (where 1 is mild, not causing distress or affecting your daily routine, and 5 is very severe, causing marked distress and/or disrupting your daily activities)?	_______/5	
9	Have you experienced any of the side-effects you have described above PRIOR to starting metformin?	Yes	−5
		No	0
	If yes, whilst taking metformin have the symptoms:	Improved	−1
		Worsened	1
		Stayed the same	0
10	Would you describe yourself as:	Tolerant of metformin treatment	0
		Mildly intolerant of metformin treatment	1
		Intolerant of metformin treatment	2
		Severely intolerant of metformin treatment.	3
For clinical staff only			
No	Item	Record	
1	Total score (0–50)	____/50	
2	Tolerability rating	(T/MI/I/SI)	
3	Patient-reported tolerability	(T/MI/I/SI)	
4	Correlation	(Y/N)	
Scoring criteria			
No	Total score range:	Record	
1	0–10: (tolerant, *T*)		
2	11–20: (mildly intolerant, MI)		
3	21–30: (intolerant, I)		
4	31–50: (severely intolerant, SI)		

#### 2.7.3 Secondary efficacy outcomes and assessment time points

Secondary outcomes include stool characteristics, bowel urgency, glucose and insulin metabolism parameters, inflammatory cytokines, and gastrointestinal function markers. Sample collection follows SOPs. Blood samples ≥3 ml are aliquoted, stored short-term at 4 °C, and long-term at −80 °C, with strict adherence to kit instructions and quality control procedures.

##### 2.7.3.1 Bristol stool form scale (BSFS)

**Figure 3:**
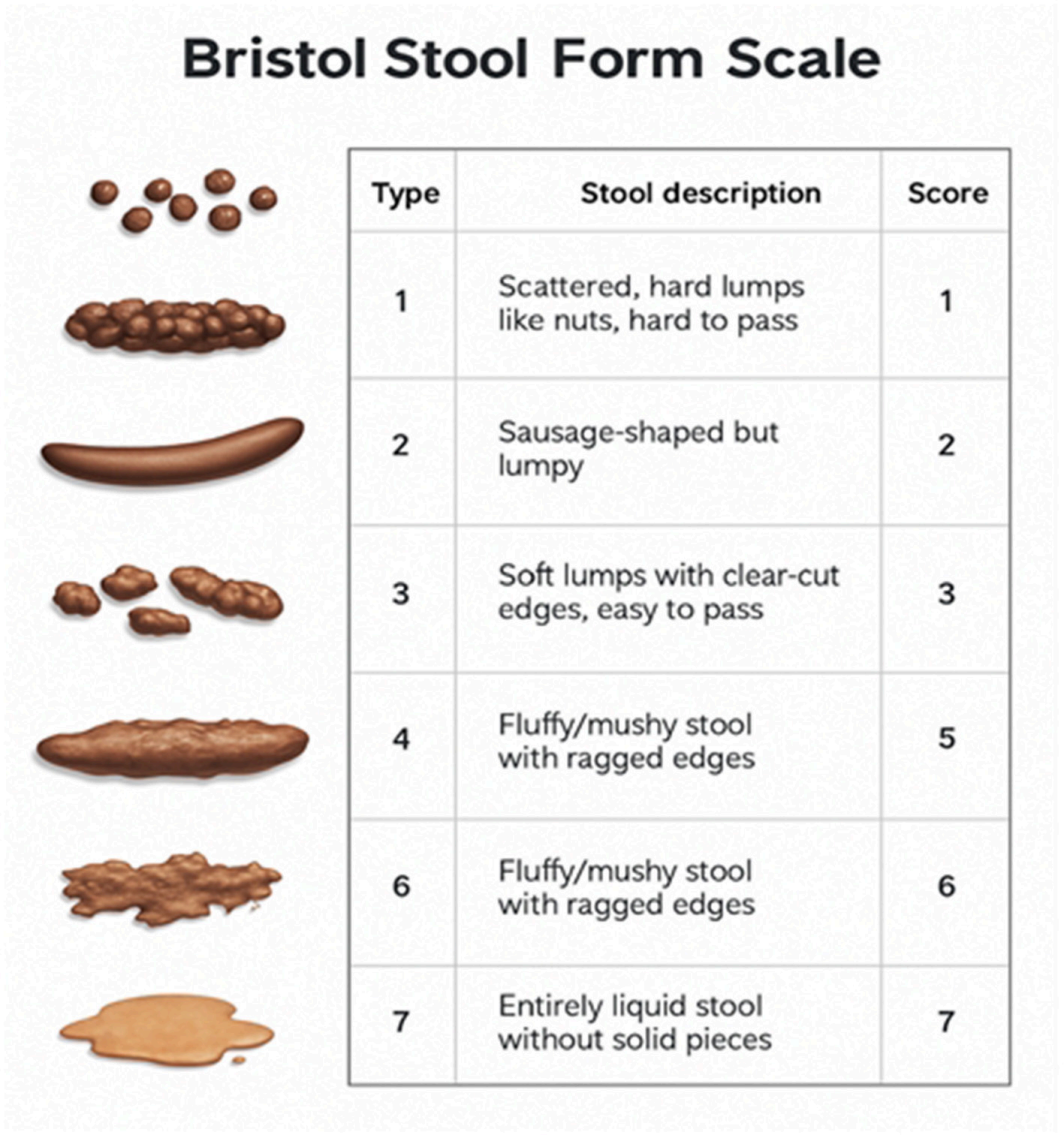
Bristol stool form classification diagram.

Stool form is evaluated using the Bristol Stool Form Scale. Participants record daily bowel movements, noting type (1–7). Weekly average stool scores are calculated to assess diarrhea incidence. If multiple types occur on the same day, the highest score is recorded. Assessment time points are baseline, Week 2, and Week 4. See Figure 3 for illustrative diagram and Table 3 for scoring criteria.

**Table 3:** Bristol stool form classification scoring table.

Type	Form	Score
1	Separate hard lumps, like nuts, difficult to pass	1
2	Sausage-shaped but lumpy	2
3	Sausage-shaped with cracks on surface	3
4	Sausage or snake-shaped, smooth and soft	4
5	Soft blobs with clear edges, easy to pass	5
6	Fluffy pieces with ragged edges, mushy	6
7	Watery, no solid pieces	7

##### 2.7.3.2 Bowel urgency

Bowel urgency reflects the temporal urgency for defecation following medication. The shortest daily response interval is recorded and quantified on a 0–3 scale: 0 = no urge/>3 h, 1 = ≤ 3 h, 2 = ≤ 2 h, 3 = ≤ 1 h. Assessment time points are baseline, Week 2, and Week 4.

##### 2.7.3.3 Glucose and insulin metabolism parameters

Parameters include fasting blood glucose (FBG), 2-h postprandial glucose (PG2h), fasting C-peptide, fasting insulin (FINS), HOMA-IR, and insulin sensitivity index (ISI). Glucose profiles are obtained via continuous glucose monitoring, and glycated albumin (GA) is used for short-term glycemic control assessment. Assessments occur at baseline and Week 2, with glucose monitoring continued for 4 weeks. HOMA-IR = (FBG × FINS)/225; ISI = 1/(FBG × FINS).

##### 2.7.3.4 Serum inflammatory markers

Serum cytokines (IL-6, IL-10, TNF-α) reflect systemic inflammation. Fasting venous blood (3–5 ml) is centrifuged at 3,500 rpm for 10 min to isolate serum and stored at −80 °C. ELISA is used for detection with *R*^2^ ≥ 0.99 for linearity. Coefficient of variation for replicate samples < 15%, and control results must meet kit specifications. Assessment at baseline and Week 2.

##### 2.7.3.5 Gastrointestinal function and metabolic serum markers

Markers include GLP-1, bile acids, 5-HT, D-lactate, diamine oxidase, and fasting insulin. Sample collection and processing follow the same protocol as inflammatory markers. ELISA is used with identical quality control and optical density requirements. Assessment at baseline and Week 2.

#### 2.7.4 Safety assessments

Safety evaluations include complete blood count, liver function (ALT, AST), and renal function (urea, creatinine). Assessment at baseline and Week 2.

### 2.8 Safety monitoring and adverse event management

#### 2.8.1 Recording and reporting of adverse events

All AEs and serious adverse events (SAEs) must be thoroughly documented in case report forms (CRF/eCRF), including event name, onset/offset dates, severity, interventions, clinical outcome, and investigator-assessed causality with study intervention. Investigators should provide prompt treatment upon identification of an AE and complete initial documentation. SAEs must be reported to the principal investigator and sponsor within 24 h and submitted to the ethics committee and regulatory authorities as required. Subjects who discontinue due to AEs should be followed until resolution, and outcomes recorded.

#### 2.8.2 Management of intervention- and drug-related AEs

Common adverse reactions related to taVNS include local pruritus, burning sensation, erythema/bruising, transient dizziness, or device displacement. Upon occurrence, stimulation should be immediately discontinued and participant status assessed; parameters may be adjusted or treatment terminated if necessary. Device malfunctions should be reported following the device adverse event protocol.

For metformin-related gastrointestinal intolerance (e.g., diarrhea, nausea, bloating), management should be guided by severity, including symptomatic support, dose adjustment, or switching to extended-release formulations. Severe dehydration, electrolyte imbalance, or life-threatening complications require immediate drug discontinuation and SAE management. All interventions (outpatient care, medication adjustments, hospitalization, referrals) must be documented and followed until resolution.

#### 2.8.3 Management of clinical deterioration

In the event that a participant experiences life-threatening conditions or requires hospitalization, all study-related procedures should be immediately suspended and emergency care initiated. The principal physician will assess the condition and determine whether continued participation is feasible.

For major safety events, the study principal investigator shall convene the DMC and Ethics Committee to conduct risk assessments and decide, based on independent recommendations, whether the trial should be paused or terminated.

### 2.9 Safety evaluation

All AEs will be graded and analyzed according to standardized systems. For practical purposes, a simplified grading mapped to the CTCAE is applied as follows:

Grade 1 (Mild): Minor symptoms requiring no intervention or only simple supportive care.

Grade 2 (Moderate): Requires outpatient management or limitation of daily activities.

Grade 3 (Severe): Requires hospitalization or prolongation of hospitalization.

Grade 4 (Life-threatening): Requires emergency intervention or life support.

Grade 5: Death directly related to the AE.

Safety endpoints include incidence, severity distribution, onset timing, intervention-related causality of AE/SAE, and laboratory abnormalities (CBC, liver and renal function). Results are primarily descriptive, with between-group comparisons performed as appropriate. De-identified individual case information may be disclosed.

### 2.10 Statistical methods

All data will be managed according to GCP guidelines for clinical trials. Two independent researchers will perform double data entry for all variables, including bowel urgency scores, glucose and insulin metabolism markers, serum inflammatory markers, and safety parameters. Cross-verification is mandatory, and the database will be locked without authorization.

Statistical analyses will be performed by blinded biostatisticians using SAS 9.4 and R 4.2.1. Two analysis sets will be applied: the intention-to-treat (ITT) set includes all randomized participants, and the per-protocol (PP) set includes participants completing ≥80% of the treatment course without major protocol violations, for sensitivity analysis.

Baseline characteristics will be summarized by group. Continuous variables are assessed for normality using the Shapiro–Wilk test: normally distributed variables are presented as mean ± SD, non-normal as median [IQR]; variance homogeneity is assessed via Levene's test. Categorical variables are expressed as counts (*n*) and percentages (%). Between-group comparisons: independent *t*-test or Mann–Whitney *U*-test for continuous variables, Pearson χ^2^ or Fisher's exact test for categorical variables.

Primary and continuous outcome variables (bowel urgency score, FBG, PG2h, FINS, C-peptide, HOMA-IR, ISI, GA, serum IL-6/IL-10/TNF-α, GLP-1, 5-HT, D-lactate, diamine oxidase, and fecal bile acids) will be analyzed using linear mixed-effects models (fixed effects: group, time, interaction; random effect: participant), reporting least-squares means with 95% *CI*. The exact mathematical formulas and covariance structures for these linear mixed-effects models will be fully reported in the final trial publication. Categorical outcomes are analyzed via χ^2^ or Fisher's exact test.

A Multiple Imputation (MI) model will be used as the primary method for missing data. The imputation model will include all primary and secondary outcomes, plus baseline covariates (age, sex, duration of diabetes, baseline metformin dose, baseline Metformin Symptom Severity Score score), with 20 Markov Chain Monte Carlo iterations to generate 20 imputed datasets. The pre-specified linear mixed-effects model will be performed on each dataset, with results pooled via Rubin's rules (Heymans and Twisk, 2022).

Complete case analysis will be conducted as a sensitivity analysis to verify the robustness of the core findings. Exploratory subgroup analyses stratified by age, sex, and diabetes duration are descriptive only.

The success of blinding will be evaluated using the James BI for all participants and independent outcome assessors at Week 2 and Week 4. The BI is scaled from 0 (all participants guessed correctly) to 1 (all participants guessed incorrectly), with 0.5 indicating completely random guesses (the standard for successful blinding). Weights are assigned consistent with the original James method: 0.00 for correct guesses, 1.00 for incorrect guesses, and 0.50 for uncertain/unable to judge responses, with uncertain responses split equally between correct and incorrect guesses. The BI and its 95% CI will be calculated using the jack-knife procedure recommended in the original method (James et al., 1996).

Safety parameters (CBC, ALT, AST, urea, creatinine) are analyzed similarly, comparing abnormality rates. All tests are two-sided with α = 0.05; effect sizes are reported with 95% CI.

### 2.11 Quality control

To ensure data completeness, accuracy, and traceability, all personnel undergo standardized SOP training before study initiation, covering: subject screening/enrollment, structured informed consent, intervention procedures, biosample collection/processing, AE/SAE documentation, and eCRF completion.

Independent CRAs will conduct regular on-site and remote monitoring per monitoring plan, including verification of informed consent, eligibility, CRF/eCRF entries, AE/SAE reporting, and biosample management. Monitoring reports and corrective actions are documented.

An independent DMC will review safety data periodically and provide risk management recommendations. All raw data are preserved as source; database is locked post-approval, with any pre-lock changes documented.

Electronic data are encrypted and backed up regularly; paper documents are stored in secure cabinets. Biosample collection, transport, aliquoting, and long-term storage follow SOP.

### 2.12 Ethics and dissemination

The study protocol has been approved by the Ethics Committee of Wangjing Hospital, China Academy of Chinese Medical Sciences (Approval No.: WIEC-YJS-2025-003-P002). This trial has been registered in the International Traditional Medicine Clinical Trial Registry (Registration No.: ITMCTR2025001086) prior to participant enrollment, in full compliance with ICMJE requirements for clinical trial registration.

Written informed consent will be obtained from all participants by trained coordinators, including study purpose, procedures, potential benefits/risks, confidentiality, and withdrawal rights.

All AEs/SAEs must be reported within 24 h. The DMC will review safety data and may recommend trial suspension or termination. Results will be disseminated via peer-reviewed journals and conferences using de-identified or aggregated data. Individual data or biosample sharing requires prior Ethics Committee and DMC approval under a data/sample use agreement. Participants will receive a plain-language summary, and key points will be posted on the hospital website.

## 3 Discussion

This protocol addresses metformin-associated gastrointestinal intolerance, a key clinical limitation in type 2 diabetes management, and aims to explore the therapeutic potential of taVNS in improving drug tolerability and optimizing metabolic control. Although metformin remains the first-line treatment, GI AEs significantly compromise adherence and long-term efficacy (Florez et al., 2010). Prior studies have indicated that metformin-induced gastrointestinal discomfort is closely linked to intestinal inflammation, mucosal metabolic dysregulation, abnormal bile acid reabsorption, and imbalances in serotonin and GLP-1 levels (Dujic et al., 2016; Ramos et al., 2016). These findings underscore the highly integrated nature of the neuro-immune-metabolic axis in the gut. Considering the vagus nerve's central role in gastrointestinal motility, secretion, and inflammation, we hypothesize that taVNS could activate the auricular–vagus–brainstem–gut reflex pathway, restore local neuroimmune homeostasis, and alleviate drug-induced intolerance. Furthermore, we postulate that taVNS, as a non-pharmacological neuromodulatory intervention, may achieve dual optimization of gastrointestinal function and glycemic control via central vagal nuclei and downstream brain–gut–metabolic networks (He et al., 2013). This would provide a novel approach to addressing the “efficacy–tolerability dose paradox” of metformin and highlight the potential clinical utility of taVNS in managing drug-related adverse effects in metabolic disorders.

Mechanistically, the effects of taVNS extend beyond peripheral modulation of gastrointestinal nerves. It likely activates central vagal circuits, including the nucleus tractus solitarius (NTS), dorsal motor nucleus of the vagus (DMV), and locus coeruleus, thereby influencing thalamo–hypothalamic integrative networks and coordinating gastrointestinal motility, mucosal barrier integrity, and metabolic signaling (He et al., 2013). Previous studies have demonstrated that taVNS promotes endogenous acetylcholine release, activates cholinergic anti-inflammatory pathways, and improves the gastrointestinal mucosal microenvironment, mitigating drug-induced intestinal hyperreactivity. taVNS may rebalance autonomic tone, with the potential to ameliorate metformin-induced gastric emptying delay and dysmotility, and stabilize pharmacokinetics, allowing patients to maintain glycemic control without dose reduction (The Wingate Institute of Neurogastroenterology TBI, 2020). This would create a novel balance between detoxification and potentiation, providing a feasible neuromodulatory strategy for comprehensive T2DM management.

Although this clinical study will focus on gastrointestinal symptoms and glycemic outcomes, the systemic effects of taVNS likely involve broader molecular and metabolic pathways. Vagal modulation is closely linked with gut microbiota homeostasis, affecting short-chain fatty acid and bile acid metabolism and intestinal barrier integrity, which may indirectly influence drug response and metabolic regulation (Zhang et al., 2025). Confirmation of these effects would provide mechanistic depth to our clinical findings and establish a basis for a multilevel central–peripheral–microecological regulatory model.

From a translational perspective, this study expands the application of taVNS to drug-related gastrointestinal intolerance, distinguishing it from prior research that primarily focused on postoperative nausea or functional dyspepsia. If the findings support our hypothesis, it would demonstrate the multidimensional potential of taVNS in modulating adverse drug reactions. Furthermore, if these findings are replicated in future multicenter trials, taVNS may become a feasible adjunctive strategy for T2DM management, particularly during high-dose metformin therapy, offering a non-pharmacological, low-side-effect approach to optimize tolerability.

Nevertheless, several limitations exist. The single-center randomized design, while enhancing intervention consistency and internal validity, may limit generalizability due to population and regional characteristics. The non-invasive nature of taVNS may introduce expectation bias, even with sham stimulation controls outside the vagal territory. Due to the anatomical specificity of the stimulation sites, blinding intervention administrators was unfeasible, potentially introducing operational bias. To mitigate this risk, we implemented strict segregation of duties and standardized protocols, ensuring that participants, outcome assessors, and data analysts remained fully blinded throughout the trial. Moreover, this clinical study will focus primarily on clinical endpoints and will not directly verify central vagal pathways or gut–brain signaling at the human level. Future investigations combining functional magnetic resonance imaging (fMRI), neural electrophysiology, and multi-omics analyses could further elucidate the central circuit mechanisms of taVNS and its interaction with drug pharmacokinetics.

In summary, this protocol is designed to evaluate whether taVNS may be a feasible and dual-modulatory intervention for metformin-associated gastrointestinal intolerance. Through synergistic effects on central vagal circuits and peripheral metabolic regulation, taVNS may simultaneously optimize gastrointestinal function and glycemic control. Integrating subsequent animal metagenomic and metabolomic data may further clarify the gut–brain–metabolic interplay underlying taVNS and metformin interactions. If validated, this mechanism would support taVNS as an effective, standardized neuromodulatory strategy within integrated T2DM management, offering new evidence for combined Chinese and Western interventions in metabolic disorders.
